# Detecting Parkinson’s Disease from Wrist-Worn Accelerometry in the U.K. Biobank

**DOI:** 10.3390/s21062047

**Published:** 2021-03-14

**Authors:** James R. Williamson, Brian Telfer, Riley Mullany, Karl E. Friedl

**Affiliations:** 1 Lincoln Laboratory, Massachusetts Institute of Technology, Lexington, MA 02421, USA; telfer@ll.mit.edu (B.T.); Riley.Mullany@ll.mit.edu (R.M.); 2 U.S. Army Research Institute of Environmental Medicine, Natick, MA 01760, USA; karl.e.friedl3.civ@mail.mil; 3 Department of Neurology, University of California, San Francisco, CA 94143, USA

**Keywords:** in-the-wild, Parkinson’s disease, wearable accelerometers, U.K. Biobank

## Abstract

Parkinson’s disease (PD) is a chronic movement disorder that produces a variety of characteristic movement abnormalities. The ubiquity of wrist-worn accelerometry suggests a possible sensor modality for early detection of PD symptoms and subsequent tracking of PD symptom severity. As an initial proof of concept for this technological approach, we analyzed the U.K. Biobank data set, consisting of one week of wrist-worn accelerometry from a population with a PD primary diagnosis and an age-matched healthy control population. Measures of movement dispersion were extracted from automatically segmented gait data, and measures of movement dimensionality were extracted from automatically segmented low-movement data. Using machine learning classifiers applied to one week of data, PD was detected with an area under the curve (AUC) of 0.69 on gait data, AUC = 0.84 on low-movement data, and AUC = 0.85 on a fusion of both activities. It was also found that classification accuracy steadily improved across the one-week data collection, suggesting that higher accuracy could be achievable from a longer data collection. These results suggest the viability of using a low-cost and easy-to-use activity sensor for detecting movement abnormalities due to PD and motivate further research on early PD detection and tracking of PD symptom severity.

## 1. Introduction

Parkinson’s disease (PD) is a chronic movement disorder that serves as a prime research candidate for early detection and later disease management technologies based on wearable accelerometry. PD offers a rich source of characteristic movement abnormalities such as tremor, gait characterized by slow movement and short stride, movement during the REM period of sleep (REM sleep behavior disorder), daytime sleepiness, and early measurable dysfunction in the magnitude and symmetry of arm swing [1,2]. Many of these symptoms are important to PD patient quality of life. As the prevalence of “sensored individuals” in the Internet of Things environment increases, data extraction methods to detect abnormal movement over time will likely provide important earlier signals of impending PD. Earlier detection, along with improved tracking of symptom severity, is likely to provide better opportunities for more effective mitigation and slowing of disease progression [3].

Simple wrist-worn accelerometry is becoming ubiquitous with the rapid proliferation of smart watches that contain this measurement capability. Early detection and management of PD might be feasible using only this measure. This is suggested by earlier PD studies using only sEMG and triaxial accelerometry worn on the affected limbs in unscripted, but closely monitored patient activity to assess tremor and dyskinesia [4]. Even in the form of a simple sports watch, a reduction in the activity and intensity of activity has been demonstrated in PD patients compared to healthy active subjects [5]. The Parkinson@ Home Study demonstrated long-term (6–12 weeks) compliance in the use of a smart watch coupled with a smartphone intended to record and monitor activity and disease-related events [6]. More recently, useful accelerometry data from unscripted free living has been reported from this Parkinson@Home Study using sensors on wrists, ankles, and back [7]. Another study that is underway, the Personalized PD Project, is collecting smartwatch multimodal sensor data on 650 PD subjects for two years [8]. Exploration of more fundamental underlying biological patterns of motion is needed to systematically classify human activity to distinguish health and disease.

The contributions of this paper are in three areas: (1) population-level early detection of PD, (2) application of signal processing techniques that are novel for PD detection, and (3) leveraging the large U.K. Biobank accelerometry data collection to gain insights into a variety of movement disorders, including PD, and other health conditions. These contributions are introduced in the following paragraphs. For population-level early detection of PD, it is desirable to use sensors that are commonplace in the population, namely smartwatches and smartphones. The analytics should be able to operate on smartwatch accelerometry independent of a most-affected side and not require that the smartwatch be worn on a dominant or non-dominant side. For initial screening, the analytics should operate on passive, free living sensing. Of course, if passive monitoring suggests further screening, scripted activities or an app questionnaire could be the next step in the screening process before being referred to a clinician. The U.K. Biobank accelerometry data set [9] has several desirable attributes for exploring the potential of early PD detection: it is large (100,000 subjects), includes subjects diagnosed with PD, as well as healthy and not diagnosed, has a relatively long duration (seven days, 24 h/day per subject), passively monitors free living subjects, provides raw accelerometry sampled at 100 Hz at the wrist, and has the accelerometer worn on the dominant wrist rather than on the wrist showing the largest PD movement symptoms. For this initial evaluation, we analyze accelerometry from 409 total subjects, including 218 PD subjects, for a total of 69,000 h of accelerometry.

This significantly exceeds the two largest data analyses reported in recent publications, namely 129 PD subjects used to create a PD severity model based on smartphone tasks [10] and 79 total subjects including 44 PD subjects and 32,000 h of data [11]. The data sets for other recent analyses are much smaller, ranging from 6 to 8100 h [12,13,14,15,16,17]. This paper is also the first analysis of PD detection from passive free living data with a large data set. Other recent work analyzing free living data has included scripted activities as part of those collections [10,11]. The larger and carefully curated PD-specific free living data sets from Parkinson@home [7] and the Personalized PD Project [8] provide opportunities for even larger scale analyses.

The early detection method considered herein is holistic and is based on all collected accelerometry containing segments of gait and low-acceleration movements. This is an important distinction from other analyses of accelerometry, which are designed to detect particular features such as tremor, bradykinesia, and gait freezing [18]. In a novel approach for PD detection, we investigate the use of correlation-structure analysis techniques that were originally developed for detecting changes in EEG functional connectivity to predict epileptic seizures [19] and that have since been shown to be advantageous for several biomedical applications [20,21,22,23,24,25,26].

The accelerometry data are segmented into activity types, extracting appropriate movement measures from each type. Specifically, we automatically assigned activity labels of gait and low-movement (LM) to segmented subsets of the data. Measures of movement dispersion were extracted from gait segments and measures of movement dimensionality from LM segments. These measures, along with the rates of occurrence of these two activity classes, were then used for PD detection.

A third contribution of this paper is to provide an example of how the U.K. Biobank accelerometry data set can be used to analyze a variety of movement disorders and health conditions through advanced analysis techniques. Thus far, the U.K. Biobank accelerometry has been used in epidemiological investigations to analyze population-level physical activity and sleep and correlations to disease [27,28,29,30,31,32,33,34], but it has not been used to explore early detection algorithms, detection and characterization of symptoms, and other disease-specific applications. Since the ICD-10 (International Classification of Diseases, Tenth Revision) codes are recorded for each subject, these types of advanced analyses can be performed for many health conditions, beyond PD.

## 2. Related Work

Our focus is on early detection of PD in free living as opposed to clinical environments. Metrics for early detection include sensitivity and specificity or the area under the receiving operating characteristic curve (AUC). Much research has been done to automate scoring of standard tests in the clinic, such as the UPDRS and Timed Up and Go assessment using wearable sensors [11,12,16]. Work has also been performed to automatically detect and quantify PD symptoms, e.g., tremor, freezing, and bradykinesia, in the clinic through scripted tests using wearable sensors [6,13,15,35]. However, for related work, we consider only results from analyses of free living data because the mean values of features differ depending on whether they are measured in the clinic or in free living conditions [36,37]. It is well documented that there are statistically significant differences in certain features on average between PD and control subjects in free living environments, e.g., for gait features [36]. However, these analyses do not calculate sensitivity and specificity.

Recently, more attention has been given to monitoring PD patients in free living conditions outside of the clinic, also referred to as “in-the-wild”, using wearable sensors and smartphones [14,38]. Free living monitoring has included both active tests, in which the subject performs scripted activities according to a smartphone app [10,11], as well as passive data collection [7]. The goals of free living monitoring have included PD screening, i.e., to detect that someone has PD, as well as scoring PD severity or PD symptom severity. PD severity scoring is of particular interest to monitor the effects of medication. The focus of our work is passive monitoring to screen for PD. The related work described in this section provides recent examples of various forms and applications of free living monitoring, both passive and active. Only two of these papers allow for quantifying early detection of PD.

San-Segundo et al. developed methods to detect tremor from wrist-worn accelerometers based on annotated laboratory collections (N = 12) and weakly labeled free living collections (N = 6) of subjects with PD [14]. An AUC of 0.89 was achieved for the laboratory collections, with the highest detection accuracies for tremors lasting five minutes or more. The performance for the free living collections was more difficult to characterize because of the weak self-reported labels. Detection of the percentage of time that tremor occurred matched well with self-reported information. However, because this analysis does not include healthy controls and because performance could not be quantified in free living conditions, its results cannot be compared with ours.

Lipsmeier et al. monitored 44 PD subjects and 35 age-matched healthy controls with smartphones in free living conditions [11]. Participants completed active tests (sustained phonation, rest tremor, postural tremor, finger-tapping, balance, and gait) on average 3.5 times per week, in addition to passive monitoring. Statistically significant differences were found between all active and passive monitoring features from PD and control participants. Sensitivity and specificity were not reported. However, our analysis of the data points in a figure for the passively monitored turning speed feature [11] yielded a sensitivity of 75% at a specificity of 81% as one operating point. These values are likely optimistic because the threshold that we chose was not evaluated on an independent test set. The number of subjects (N = 79) was also small compared to our analysis (N = 409), but this performance serves as a point for comparison.

Zhan et al. monitored 129 PD subjects with smartphones over the course of six months [10]. Data were collected actively through five smartphone activities (voice, finger tapping, gait, balance, and reaction time), as opposed to the passive monitoring approach that we investigate. Machine learning was applied to develop a mobile PD score (mPDS) that correlated well with several standard tests, including the Movement Disorder Society Unified PD Rating Scale total (r = 0.81) and Part III only (r = 0.88) and the Hoehn and Yahr stage (r = 0.91). The score also improved in response to dopaminergic therapy. The mean mPDS was 47% lower in control versus PD participants, but the sensitivity and specificity were not reported, so its results cannot be compared to ours.

In another large smartphone data collection, the mPowerstudy [39] included 1087 participants who self-reported as having a professional diagnosis of PD and 5581 participants who did not. Scripted activities were termed “memory,” “tapping”, “voice”, and “walking”. A preliminary exploration of the ability to detect PD from a subset of the walking data was performed [40], but methodological issues prevent a comparison with our work.

Evers et al. analyzed data from 25 PD subjects and 25 controls in their homes, which were from the Parkinson@Home validation study [7]. Video recordings were manually annotated to provide truth information. Participants wore sensors with accelerometers and gyrometers at five body locations, including on each wrist. A classifier was developed to distinguish between PD (pre-medication) and control subjects based on spectral features extracted from gait segment, yielding AUCs of 0.75 for the most affected wrist and 0.49 for the least affected wrist. Classification using sensors worn at the ankles and pants pocket did not yield higher AUCs. In contrast, in the U.K. Biobank data that we analyze, subjects were asked to wear the accelerometer on their dominant wrist, which is more appropriate for early detection, since the most affected wrist would not necessarily be known. However, since AUCs for both wrists were reported, this work provides a good comparison point with ours.

Raykov et al. analyzed the same Parkinson@Home data set to classify whether gait segments corresponded to times before or after medication [41]. They achieved a classification accuracy that was comparable to using annotations from the video recordings. This classification application is different from early detection and thus cannot be compared.

## 3. Materials and Methods

**Figure 1:**
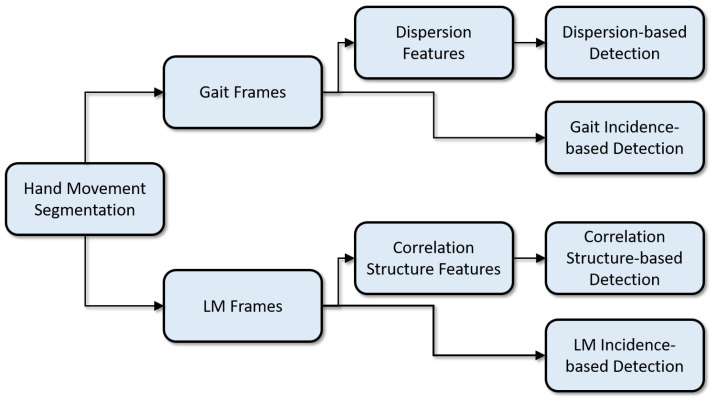
Processing pipeline for PD detection.

This section describes the U.K. Biobank data and the PD detection algorithm. It describes how wrist-worn accelerometry data were segmented into two movement types (gait and LM), how frame-level features were extracted from each movement type, and how these features, along with frame incidence levels, were used to detect PD. The PD detection processing pipeline is illustrated in Figure 1.

### 3.1. Subject Population

The U.K. Biobank [42] data set includes more than 500,000 individuals, with a subset of 100,000 participants who wore an Axivity (Newcastle upon Tyne, U.K.) AX3 accelerometer data logger on the dominant wrist for one week. All subjects provided their informed consent for participation in the U.K. Biobank survey and agreed to further analyses of their anonymized data; these data are generally available to qualified researchers [42]. Initial recruitment of this longitudinally studied cohort was performed in 2006–2010 and included sampling from a wide demographic of U.K. 40–69 year olds. The data collection and methodology have been previously described [42]. Health data were obtained from health record linkages, primarily to National Health System datasets, and included expert-led adjudication of disease outcomes, including neurodegenerative disease diagnoses [42]. The accelerometry substudy was conducted between 2013 and 2015 using the wrist-worn accelerometer; participants were asked to wear the device on their dominant arm for one week of data recording [9].

The Axivity device recorded at 100 Hz with a range of ±8 g. We selected as the PD population all 218 subjects with recorded accelerometry data and with a primary ICD-10 (International Classification of Diseases) code of Parkinson’s disease (G20). As a healthy control population, we selected 191 age-matched subjects with accelerometry data who did not have movement or sleep disorders (negative codes for G20–G26, G35, G47). The data from these 409 subjects (218 PD and 191 control) were initially analyzed.

Subjects were excluded from further analysis if no data segments were found with sustained gait or LM activity (see Section 3.2). This resulted in 380 subjects (202 PD and 178 control) used in the analysis described in this paper. PD subjects (62.4±5.8 years) and control subjects (61.7±6.1 years) were age matched. There was an expected overrepresentation of male subjects among the PD population (131 male PD, 83 male control) compared to female subjects (71 female PD, 95 female control). Therefore, the final classification results are also reported for males and females separately (Section 4.2) to ensure that detection performance is not confounded by gender.

### 3.2. Gait and LM Segmentation

The analysis approach depends on first segmenting data based on the detection of two types of sustained activity and then extracting features from data frames within the activity segments. Similar methods of activity-based segmentation have been used previously to process walking and running gait from torso accelerometry for estimating load carriage [20] and exertional heat stroke risk [43]. Activity segments were included only if they were of sufficient duration to indicate a sustained, regular activity from which reliable features could be extracted. The first activity type is gait, which is characterized by a high magnitude of average accelerations, with periodicities in each axis that are in a plausible range of arm swinging rates during walking. The second behavior type is LM, which is characterized by a range of lower magnitude average accelerations with no constraints on periodicity. Different feature analysis techniques (Section 3.4 and Section 3.5) were applied to data segments from each activity type.

Accelerometry data consisted of 3 axis accelerations, $x\left(t\right)=\{x_{1}\left(t\right),x_{2}\left(t\right),x_{3}\left(t\right)\}$, in units of g, from a wrist-worn accelerometer (sampling frequency 100 Hz, dynamic range ±8 g). Behavioral categories were initially segmented from the acceleration magnitude signal,

(1) $$m\left(t\right)={\left[x_{1}{\left(t\right)}^{2}+x_{2}{\left(t\right)}^{2}+x_{3}{\left(t\right)}^{2}\right]}^{0.5}.$$

The local acceleration energy, represented by the standard deviation of the acceleration magnitude, was computed in a sliding window with a duration $\tau_{1}=10$ s,

(2) $$\sigma_{m}\left(t\right)={\left[\frac{1}{N(t)}\sum_{t^{\prime }\in W\left(t\right)}{\left(m\left(t^{\prime }\right)-\mu_{m}\left(t\right)\right)}^{2}\right]}^{0.5},$$

where $t^{\prime }\in W\left(t\right)$ if $|t-t^{\prime }|\leq \tau_{1}$. The window count and windowed mean were computed as follows:

(3) $$N\left(t\right)=\sum_{t^{\prime }\in W\left(t\right)}1.$$

(4) $$\mu_{m}\left(t\right)=\frac{1}{N(t)}\sum_{t^{\prime }\in W\left(t\right)}m\left(t^{\prime }\right),$$

Gait segments were labeled wherever contiguous values of $\sigma_{m}\left(t\right)$ were a suprathreshold, $\sigma_{m}\left(t\right)>\Gamma_{1}$, across a $\tau_{2}=30$ s interval. However, strict contiguity of suprathreshold values within gait segments was not required, as short subthreshold gaps were tolerated if their duration was $\tau_{3}=15$ s or less. LM segments were labeled wherever contiguous values of $\sigma_{m}\left(t\right)$ were between two thresholds, $\Gamma_{2}<\sigma_{m}\left(t\right)<\Gamma_{3}$, across a $\tau_{4}=240$ s interval.

### 3.3. Gait and LM Frames

Gait and LM segments were divided into contiguous 10 s frames, and features were then extracted independently from each frame, as described in Section 3.4 and Section 3.5. The gait frames were required to contain a minimal amount of gait regularity, as quantified by acceleration periodicity within a range of plausible gait periods.

In each gait frame, autocorrelations of the 10 s acceleration signals in each axis were computed. The first positive autocorrelation peak was hypothesized to represent the average step time duration, which corresponded to the time it took for the arm to swing from back to front or from front to back. Autocorrelation peaks were considered within a range of allowed time delays between $\tau_{6}=0.2$ s and $\tau_{7}=1.75$ s and were additionally only considered for time delays greater than than the first negative autocorrelation within that time range. Finally, the autocorrelation peak height in all three axes was required to be greater than $\Gamma_{4}=0.1$.

Table 1 summarizes the parameters governing the segmentation of acceleration signals into gait and LM behavior categories and then the labeling of valid gait and LM frames for further feature processing. These parameters were selected in an initial exploratory analysis of 69 subjects (40 PD and 29 control) and held fixed thereafter.

**Figure 2:**
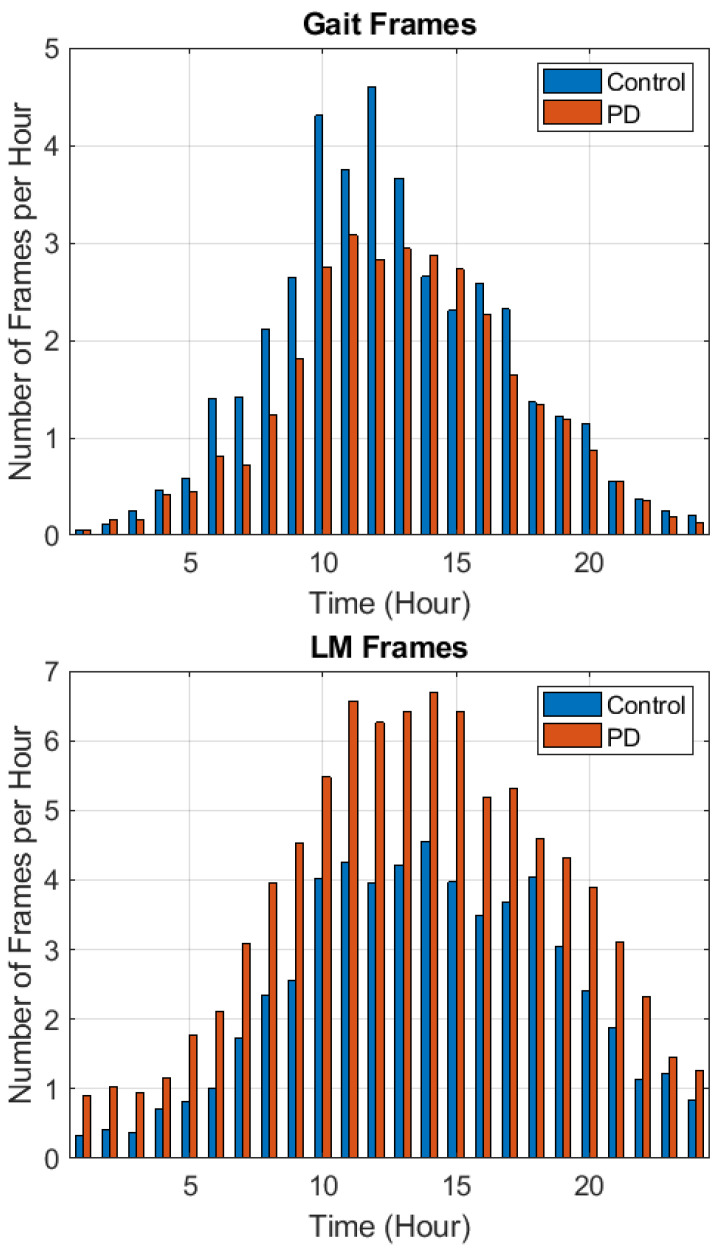
**Top**: Average number of detected gait frames per hour per subject for control and PD subjects. **Bottom**: Average number of detected low-movement (LM) frames per hour per subject for control and PD subjects.

Differences were found between control and PD subjects in the incidence (the number of frames per day) of gait and LM frames. Therefore, gait and LM frame incidences were also used as features for PD detection, in addition to accelerometry-based features extracted from gait and LM frames. Figure 2 shows (following the full seven days of data collection) the incidences of gait (top) and LM (bottom) frames for the control and PD populations on an hourly basis. Gait frames were most common between late morning and mid afternoon (10 AM to 4 PM). PD subjects typically had a smaller number of gait frames than control subjects. LM frames were most common between 10 AM and 6 PM. PD subjects typically had a larger number of LM frames than control subjects.

**Figure 3:**
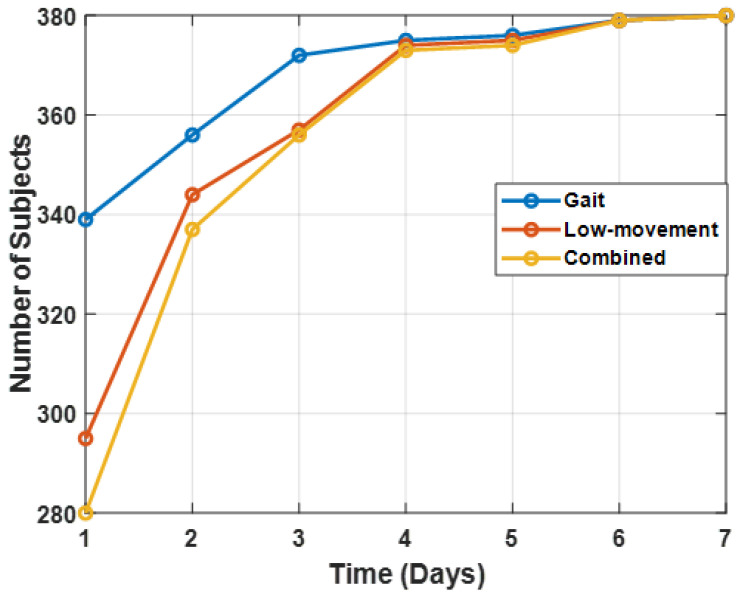
Number of subjects for whom gait frames, LM frames, or both types of frames were detected.

Figure 3 illustrates the evolution, over the seven days of the data collection, of the total number of subjects that had a positive number of gait frames, LM frames, or both. Gait frames were detected in a larger number of subjects than LM frames in the first three days of the data collection. By the fourth day, gait and LM frames were detected in a roughly equal number of subjects. The number of subjects increased only a small amount in the remaining three days. As reported in Section 4.2, PD detection accuracy was evaluated as a function of the number of days analyzed (Section 3.7.3).

### 3.4. Gait Dispersion Features

It is well established that PD reduces gait regularity [44]. Dispersion is a method for quantifying gait regularity from accelerometry, independently of the magnitude of acceleration signals [43]. Dispersion, which quantifies the average distance between normalized accelerations across a frame of data, is a measure of acceleration variability. Gait signals are quasiperiodic and tend to exhibit a high degree of similarity in acceleration values in nearby steps. Dispersion indicates deviation from this tendency.

Specifically, dispersion was computed as follows. First, the acceleration values in a 10 s LM frame, *n*, were z-scored into standard units in each axis, ${\hat{x}}_{i}\left(t\right)$. Next, outlier values were removed from the analysis, as these seemed to degrade the usefulness of the dispersion feature. Let V(n) be the set of valid (i.e., non-outlier) time points in frame *n*, defined by excluding points greater than $\Gamma_{5}=2$ standard deviations in any of the three axes:

(5) $$t\in V\left(n\right)\;\mathrm{if}\;|{\hat{x}}_{i}\left(t\right)|<\Gamma_{5}\;\mathrm{for}\;i=1,2,3$$

Next, average distances between all pairs of valid values in each axis were computed. L1 distances were used. The dispersion in axis *i* is thus:

(6) $$D_{i}\left(n\right)=\frac{1}{S(n)}\sum_{t_{1},t_{2}\in V\left(n\right)}\left|{\hat{x}}_{i}\left(t_{1}\right)-{\hat{x}}_{i}\left(t_{2}\right)\right|,$$

where:

(7) $$S\left(n\right)=\sum_{t_{1},t_{2}\in V\left(n\right)}1.$$

The dispersion feature vector for the *n*th frame is therefore the three-element vector $D\left(n\right)=\{D_{1}\left(n\right),D_{2}\left(n\right),D_{3}\left(n\right)\}$.

### 3.5. LM Correlation Structure Features

We hypothesized that PD also causes changes in movement dynamics during LM periods, during which the average magnitude of wrist movements is relatively small. Dynamics may differ due to tremor, rigidity, and bradykinesia [15], resulting in differences in movement dimensionality across a range of movement frequencies. For a particular range of movement frequencies, dimensionality can be quantified by the fraction of total movement variance that is explained by correlations between acceleration signals, using a spacing of relative time delays that is appropriate for the range of movement frequencies.

A general approach was developed that quantifies movement dimensionality in this way, using correlation patterns among multichannel feature sets. This approach has been used in many studies to detect and estimate alterations in neuromotor coordination from speech [23,24,25,26], including motor and cognitive symptoms due to Parkinson’s disease [21,22]. The approach has also been used for detecting alterations in torso accelerations during gait due to load carriage [20] and mild traumatic brain injury [25], as well as for detecting alterations in hand movements during drawing due to autism [26].

This approach uses correlation structure features, which are the eigenspectra of channel-delay correlation matrices. Here, “channel” refers to the three acceleration axes and “delay” refers to time delays at which correlations are computed both between and within acceleration axes. Furthermore, correlation matrices are constructed with time delays at multiple time delay scales. Another way to think of the matrix construction is that correlation matrices are constructed from an expanded number of acceleration time series, with the expanded number obtained via time-delay embedding of the original signals at multiple delay scales.

Specifically, a channel-delay correlation matrix at delay scale *j* is computed as:

(8) $$R_{j}=\left[\begin{array}{ccc} R_{1,1}\left(j\right) & \cdots & R_{1,M}\left(j\right)\\ \vdots & \ddots & \vdots \\ R_{M,1}\left(j\right) & \cdots & R_{M,M}\left(j\right) \end{array}\right]$$

where *M* is the number of low-level feature channels. Each submatrix $R_{c_{1},c_{2}}\left(j\right)$ contains the set of correlations between channels $c_{1}$ and $c_{2}$ at scale *j*,

(9) $$R_{c_{1},c_{2}}\left(j\right)={\left[\begin{array}{ccc} r_{1,1}\left(j\right) & \cdots & r_{1,N}\left(j\right)\\ \vdots & \ddots & \vdots \\ r_{N,1}\left(j\right) & \cdots & r_{N,N}\left(j\right) \end{array}\right]}_{c_{1},c_{2}}$$

*N* is the number of delays per channel, and ${\left[r_{d_{1},d_{2}}\left(j\right)\right]}_{c_{1},c_{2}}$ is the correlation, at scale *j*, between channel $c_{1}$ at delay $d_{1}$ with channel $c_{2}$ at delay $d_{2}$.

The eigenvalues of the correlation matrix, $R_{j}$, rank ordered from largest to smallest, quantify the correlation structure. Consistent with past practice when using correlation structure features [23,24,25], the eigenspectra feature vectors were concatenated across the four delay scales. Table 2 summarizes the parameters used to extract these features, which is the same parameterization typically used in the literature [23,24,25].

**Figure 4:**
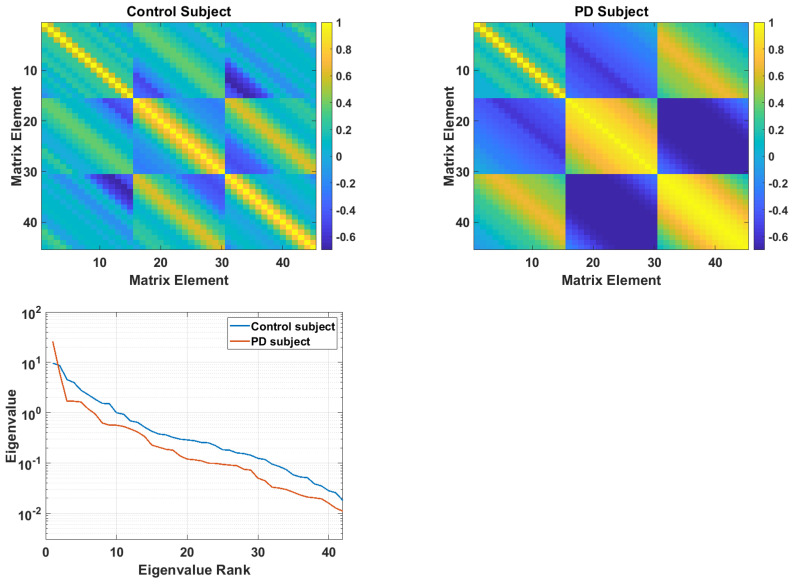
Channel-delay correlation matrix from a control subject (**top left**) and from a PD subject (**top right**). Eigenspectra from the two matrices, ranked from largest to smallest (**bottom**).

Figure 4 illustrates correlation structure matrices from a control subject (top left) and PD subject (top right) at the largest delay scale, $d_{4}$. These matrices are described in Equations (8) and (9). The eigenspectra from the two matrices are shown at the bottom. These eigenspectra reflect the general pattern that was found (see Section 4.1), in which PD subjects have larger low-rank eigenvalues, but smaller mid- and high-rank eigenvalues. This pattern shows a lower dimensionality in the acceleration time series of PD subjects.

### 3.6. Feature Effect Sizes

Effect sizes between PD and control populations were computed for all features described above. These consist of: (1) gait frame incidence; (2) LM frame incidence; (3) gait dispersion values in each axis; (4) LM correlation structure features (matrix eigenspectra) at four delay scales.

### 3.7. Classification Algorithm

#### 3.7.1. Cross-Validation and Parameter Selection

Five-fold cross-validation was used, with no mixing of subject data across training and test folds. Subjects were randomly assigned to the five test folds, with the number of PD and control subjects apportioned as equally as possible in each fold. Parameter selection was applied independently to each training fold. Parameter selection consisted of selecting the number of principal components (PCs) in each feature set. The number of PCs was selected that explained a fixed fraction of the variance (0.975) in the training set. For the gait dispersion features, this resulted in 3 PCs in all five folds. For the LM correlation structure features, this resulted in 19 PCs in four folds and 18 PCs in one fold.

#### 3.7.2. Gaussian Mixture Model

All features were input to a Gaussian mixture model (GMM) classifier. GMMs represent feature densities using a finite number, *K*, of Gaussian distributions, which are centered on *K* randomly selected data points. K=5 was selected in an exploratory analysis on a smaller data set of 69 subjects. Learning in GMMs has an unsupervised stage followed by a supervised stage. In the unsupervised stage, GMMs are trained using the expectation-maximization algorithm, which iteratively moves the Gaussians (adapting their means), changing their shape (adapting their covariance matrices) and changing their heights (adapting their weights). This was done using L=4 batch-level presentations of the training data. In this unsupervised stage, a single GMM was fit to all the training data (inclusive of all output classes). The supervised portion of training involved adapting this single GMM into two class-specific GMMs, using the non-iterative training procedure described in Reynolds et al. [45], using relevance parameter r=16.

The covariance matrices were restricted to be diagonal matrices throughout and were initialized to have large values. Given a z-scored data set with zero mean, unit standard deviation in each dimension, the initial covariance matrices were the identity matrix times the initial covariance, which was set to $\sigma_{1}^{2}=100$. Using a large spherical covariance allowed the set of Gaussians to settle into good density representations and avoided premature commitment, which can lead to them settling into bad local maxima in the likelihood space. The diagonal elements were not allowed to become smaller than $\sigma_{2}^{2}=0.01$, to prevent overfitting. Finally, the Gaussian weights (prior probabilities) were initialized to 1/*K*.

Table 3 summarizes the GMM parameters, which have standard values that have been used in multiple previous studies [24,25,26]. The GMM output when presented with a feature vector from the test data is a likelihood score for each class model. This was obtained by summing the likelihoods of the *K* Gaussians comprising each GMM class model. For gait dispersion features and LM correlation structure features, likelihoods were computed on a per-frame basis. The net class likelihood for each activity type was the mean of all GMM frame likelihoods. The PD detection score was then the log-likelihood ratio: log(likelihood of class 2) − log(likelihood of class 1).

#### 3.7.3. Fusing Detection Scores

Different feature types were fused by summing their detection scores (i.e., classifier log-likelihood ratios). This means that the scores were treated as independent probabilities. However, because the multiframe scores (from gait dispersion and LM correlation structure) were obtained by averaging likelihoods over multiple frames before computing the log-likelihood ratio, the appropriate relative weighting between these scores and the gait frame incidence and LM frame incidence scores was unclear. The weighting coefficient for combining these scores (α) was selected empirically, based on fused classification accuracy. The selected value, α=0.15, also approximately equalized the variance of the multiframe scores and the frame incidence scores.

Specifically, scores were fused as follows. Let $S_{G1}$ be the gait log-likelihood score from dispersion feature likelihoods averaged across all gait frames. Let $S_{G2}$ be the gait frame incidence log-likelihood score. Then, the total gait score is $S_{G}=S_{G1}+\alpha S_{G2}$. Similarly, let $S_{LM1}$ be the LM log-likelihood score from correlation structure feature likelihoods averaged across all LM frames. Let $S_{LM2}$ be the LM frame incidence log-likelihood score. Then, the total LM score is $S_{LM}=S_{LM1}+\alpha S_{LM2}$.

Finally, PD detection performance is reported using three measures. These measures were computed using the union of test data across all five test folds. The measures were the area under the receiver operating characteristic curve (AUC) and detection sensitivity given false positive rates (FPRs) of FPR = 0.1 and FPR = 0.2.

## 4. Results

### 4.1. Effect Sizes

**Figure 5:**
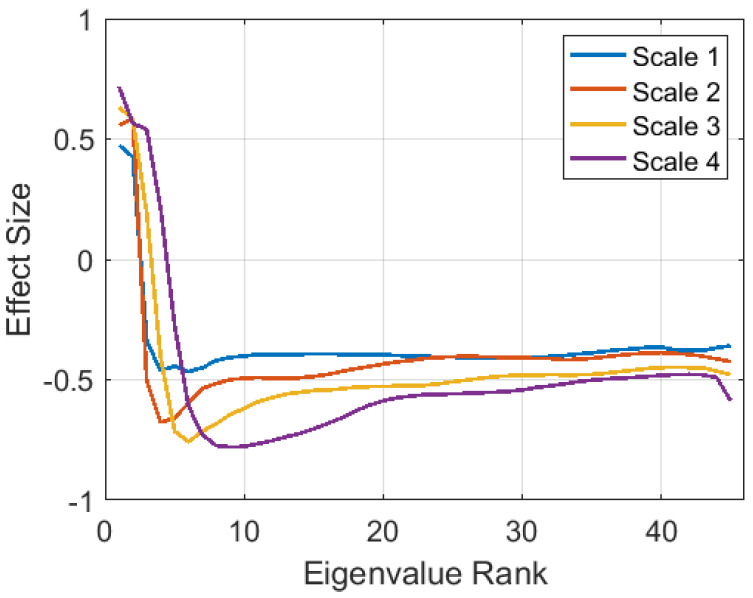
Effect sizes (computed across all subjects) of mean eigenspectra from LM frames, at four delay scales. Eigenspectra are rank ordered from largest to smallest.

The differences in the incidence of gait and LM frames per day are reflected in Cohen’s D effect sizes of −0.52 for gait frame incidence and 0.43 for LM frame incidence (Table 4). Table 4 also shows negative effect sizes for gait dispersion features in all three axes. These effect sizes were computed based on the mean within-subject dispersion values, computed across all gait frames. The effect size was smaller in the lateral direction (Axis 3) than in the first two axes, which were aligned along the plane in which the arm is typically swinging. The sign of the effects indicates less acceleration dispersion in PD subjects during gait. The effect sizes of within-subject mean eigenspectra from LM frames were also computed and are shown in Figure 5. Similar patterns occur at all four delay scales, showing lower dimensionality for PD subjects (negative effects for smaller, higher rank eigenvalues).

### 4.2. Detection Accuracy

Accuracy in detecting PD is summarized in Table 5. These results were based on the union of PD detection scores from subjects across the five test folds, using all seven days of data per subject. The first column shows different feature fusion combinations (see Section 3.7.2 and Section 3.7.3) in the first column. The area under the ROC curve (AUC) values are shown, inclusive of both genders and for males and females separately in order to verify that detection accuracy is not confounded by the skewed gender representation of PD subjects. Overall, the gender differences in accuracy were small. The highest AUC value of 0.85 was obtained by fusing all features (Row 8). The AUC results were statistically significant (p<0.01) for all feature combinations, based on both t-tests and Mann–Whitney U-tests [46]. Sensitivity levels are also shown for fixed false positive rates of FPR = 0.1 and FPR = 0.2. The feature combinations in Rows 7 and 8 produce the highest sensitivities.

**Figure 6:**
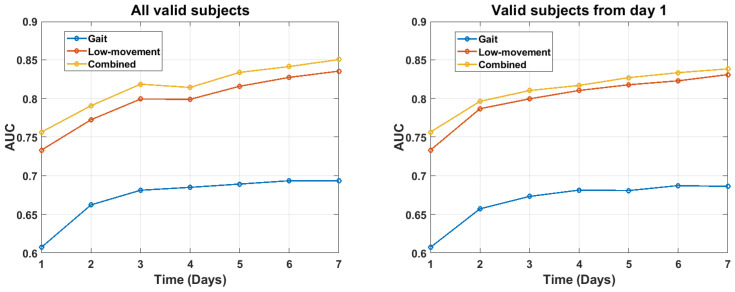
AUC values for gait features, LM features, and both features combined, as a function of the number of days of data that were used. **Left**: All subjects with valid data as of the day reported are included. **Right**: Only subjects with valid data on the first day are included.

It is also of interest how much test data were required to obtain accurate detection results. To evaluate this question, PD detection was evaluated as a function of the number of days of test data that were used. These results were computed in two different ways. First, they were plotted for the full set of subjects for whom valid gait, LM, or both frames were detected as of each time point (see Figure 6, left). The number of subjects with valid gait frames, LM frames, or both varied across the week (see Figure 6). This change in subject composition could obscure improvements in within-subject accuracy that were due to the accumulation of additional data across the week. Therefore, we also plot results for those subjects who had valid data only on the first day (see Figure 6, right). In both cases, the results were roughly the same. LM features produced higher accuracy, by a roughly constant amount, over all seven days. Fusing gait with LM features in turn produced a fixed small increment in accuracy. Total accuracy gradually increased over the full one week time course. These results suggest that higher accuracy may be attainable by collecting accelerometry data over a longer duration than seven days.

## 5. Discussion

The contributions of this paper are in three areas: (1) population-level early detection of PD using wrist-worn accelerometry that can be measured by smartwatches, (2) application of signal processing techniques that are novel for PD detection, and (3) leveraging the large U.K. Biobank accelerometry data collection to gain insights into a variety of movement disorders, including PD and other health conditions.

Novel signal processing techniques were used to measure movement dispersion during gait periods and movement dimensionality during non-gait low-amplitude movement periods. The accelerometry analysis approaches (movement dispersion and dimensionality) have been validated previously on torso-worn accelerometry data [20,25,43]. The movement dimensionality approach has also been applied to many alternative modalities, including the analysis of speech differences due to PD [21,22]. As the primary focus of this work was on activity segmentation and movement characterization, a single standard machine learning approach was adopted to quantify the effectiveness of the features in detecting PD. We found that PD subjects showed a greater incidence of sustained low-movement activity and a lower incidence of sustained gait activity than healthy controls. We also found differences in movement dynamics given these activities. For both activities, PD subjects showed a reduced and rigid movement profile relative to controls. An additional important finding was that this profile became more complete across seven days of data collection, demonstrating the value of activity sampling across multiple days.

It is difficult to compare our results with those from other analyses because there has been little work that has attempted to quantify early detection in terms of sensitivity and specificity or the receiver operating curve for detecting PD. There are two primary points of comparison. Lipsmeier (2018) analyzed free living smartphone data from 44 PD subjects and 35 healthy controls, measured from both scripted activities and passive monitoring [11]. Statistically significant differences between PD and controls were found for several scripted and passive features, but sensitivity and specificity were not reported. However, our analysis of the data points for the passively monitored turning speed feature [11] yielded a sensitivity of 75% at a specificity of 81% as one operating point. These values are likely optimistic because the chosen threshold was not evaluated on an independent test set. The number of subjects (79) was also small compared to our analysis (409). With these caveats, this operating point was similar to our fused result of 75% sensitivity at 80% specificity (Table 5, Row 8). This suggests that detection performance might be further improved by combining our approach with additional features such as reported in [11].

Another result that can be compared to ours because it used unscripted activity data is Evers et al. (2020), in which wrist-worn accelerometry from gait was analyzed [7]. They were able to detect PD versus controls with AUC = 0.75 using data from the most affected wrist and AUC = 0.49 from the least affected wrist. In comparison, in our analysis of unscripted gait data using the dominant wrist, we detected PD with AUC = 0.85 by fusing scores from gait and LM data (see Table 5).

The methods and data set used in this paper have strengths and limitations. Methodologically, a strength is that the data-driven features allow PD to be detected without explicit detection of particular symptoms (e.g., tremor, freezing, or bradykinesia) and hence without requiring symptom labeling in the training data set. Another strength is that the data set is significantly larger than others and was collected passively from free living individuals. The data were collected with the accelerometer worn on the dominant wrist (as opposed to on the most-affected wrist), which is consistent with a use case of providing early detection of PD using wrist-worn devices that subjects wear as part of their everyday routine. The feature extraction algorithms should be insensitive to the sensor wear orientation (i.e., whether it is worn at the top or bottom of the wrist). Table 4 shows similar effects for dispersion features in the three acceleration axes, and the correlation structure features are invariant to the axis identity. In addition, the algorithm does not require that the sensor be worn on the most-affected wrist.

Data set limitations include: limited duration of data collection (one week), lack of PD diagnosis by a specialist (the G20 ICD-10 code may have been recorded by a general practitioner, for example), lack of easily accessible details on PD severity, and lack of labels for particular symptoms that may be present.

There are several opportunities for future work with the U.K. Biobank dataset. One promising next step would be to investigate the false alarm cases to understand what aspects of the data appear to resemble PD. In fact, it is possible that some of the false alarms may be due to early cases of PD that have not yet been diagnosed. The PD cases can also be further investigated, to determine evidence for tremor or bradykinesia symptoms using algorithms that have been validated in the laboratory by others. Combining symptom features with the data-driven features investigated in this work may improve sensitivity and specificity. A comparative performance analysis that includes more sophisticated machined learning approaches is also merited. Another next step would be to analyze the PD subjects to identify data-driven phenotypical clusters and map those clusters to potential differences in disease severity.

The U.K. Biobank sub-study that produced these data demonstrates the feasibility of wearable accelerometry data acquisition conducted by mail with the participants. Further examination of movement patterns and validation of algorithms for the early detection of chronic disease such as Parkinson’s disease should be pursued with other longitudinal cohorts such as the Parkinson@Home study, the Personalized PD Project, and the U.S. Department of Defense Millennium Cohort (MILCO) study, with aging veterans and documented environmental and stress exposures [47].

Other future steps include validating the activity pattern differences found in this paper on newly diagnosed PD patients and using this approach to examine the progression of disease symptoms over long-duration data collections. It should also be further tested if the signal is unique to PD or if it is also manifested in other conditions that involve abnormal balance and movement components. The other conditions could include temporary impairments in brain control of motor function following a traumatic head injury or chemical insult.

Wrist-worn accelerometry could be one component of a multimodal system for PD detection and symptom severity tracking. Other measurements, such as voice analysis for speech disturbance characteristics of PD, would likely improve the specificity and sensitivity of motor and cognitive symptoms of PD [22,48], thereby providing better biofeedback and technologist assistance to patients [13]. Wearable accelerometry already has proven value in monitoring and encouraging regular exercise behaviors in the general population, and daily exercise has specific benefits for PD patients in slowing the progression of early disease and improving quality of life. Improved algorithms for more accurate scoring of wrist-worn accelerometry for sleep quality and duration in PD patients could also be a great benefit to patients and their medical providers [49].

## Figures and Tables

**Table 1:** Parameters governing the selection of gait and low-movement (LM) frames.

Parameter	Value	Description
$\tau_{1}$	10 s	Window size for local statistics
$\tau_{2}$	30 s	Minimum gait segment duration
$\tau_{3}$	15 s	Maximum gap within gait segment
$\tau_{4}$	240 s	Minimum LM segment duration
$\tau_{5}$	10 s	Gait or LM frame duration
$\tau_{6}$	0.21 s	Minimum autocorrelation peak delay
$\tau_{7}$	1.75 s	Maximum autocorrelation peak delay
$\Gamma_{1}$	0.05 g	Minimum gait $\sigma_{M}$
$\Gamma_{2}$	0.001 g	Minimum LM $\sigma_{M}$
$\Gamma_{3}$	0.03 g	Maximum LM $\sigma_{M}$
$\Gamma_{4}$	0.1 g	Minimum gait autocorrelation peak

**Table 2:** Parameters governing features extracted from gait and low-movement (LM) frames.

Parameter	Value	Description
$\Gamma_{5}$	2	Dispersion outlier threshold
*M*	3	Number of acceleration channels
*N*	15	Number of delays per channel per scale
$\left\{d_{1},d_{2},d_{3},d_{4}\right\}$	{1,3,7,15}	Delay spacing per scale

**Table 3:** Parameters governing PD classification and fusion.

Parameter	Value	Description
*K*	5	Number of GMM components
*L*	4	Number of GMM iterations
$\sigma_{1}^{2}$	100	GMM initial variance
$\sigma_{2}^{2}$	0.01	GMM minimum variance
*r*	16	GMM adaptation parameter
α	0.15	Weight for fusing frame incidence scores

**Table 4:** Effect sizes of gait and LM frame incidence and gait dispersion features.

Feature	Activity	Effect Size
Gait frame incidence	Gait	−0.52
LM frame incidence	LM	0.43
Dispersion, Axis 1	Gait	−0.57
Dispersion, Axis 2	Gait	−0.50
Dispersion, Axis 3	Gait	−0.29

**Table 5:** PD detection performance for different feature combinations given seven days of accelerometry data.

	AUC	Sensitivity
Fused Detection Scores	All, Male, Female	FPR = 0.1, FPR = 0.2
1. $S_{G1}$	0.66, 0.66, 0.66	0.33, 0.40
2. $S_{G2}$	0.66, 0.63, 0.74	0.32, 0.47
3. $S_{G1}+\alpha S_{G2}$	0.69, 0.68, 0.74	0.35, 0.52
4. $S_{LM1}$	0.83, 0.84, 0.80	0.53, 0.72
5. $S_{LM2}$	0.61, 0.56, 0.68	0.17, 0.32
6. $S_{LM1}+\alpha S_{LM2}$	0.84, 0.85, 0.82	0.63, 0.71
7. $S_{G1}+\alpha S_{G2}+S_{LM1}$	0.84, 0.85, 0.83	0.63, 0.79
8. $S_{G1}+\alpha S_{G2}+S_{LM1}+\alpha S_{LM2}$	0.85, 0.85, 0.85	0.65, 0.75

## Data Availability

These data are held by the UK Biobank (https://www.ukbiobank.ac.uk/).

## Abbreviations

The following abbreviations are used in this manuscript:

AUC	Area under the receiver operating characteristic curve
FPR	False positive rate
GMM	Gaussian mixture model
LM	Low-movement
PD	Parkinson’s disease
UPDRS	Unified Parkinson Disease Rating Scale
